# Five Letters and Replies

**Published:** 1880-01

**Authors:** 


### Our Correspondence.

PHILADELPHIA, Nov. 2, 1879.

DR. UP DE GRAFF:

I read in the current BISTOURY thy cautionary notice of Sarah Bernhardt, which I heartily endorse. Query: Will thee extend the same cautionary care in regard to *men* of that character, equally censurable, often more dangerous, who are not only received but *invited* "to enter our parlors for social intercourse, where are congregated our young men and maidens." It is time, I think, that an impartial course should be demanded in this matter.

Sincerely,

S. H. P.

The lady is right. There should be no partiality shown in the treatment of the licentious of either sex. For the same reason that society does not tolerate the presence of the courtezan, should her male patron be condemned. The one is quite as blameworthy as the other.

---

GENEVA, N. Y., Nov. 20, 1879.

DEAR BISTOURY:

Here is my dollar for last year's and the coming year's subscription. I would not be without my BISTOURY for anything. I use it as my encyclopedia of information. I have it bound for six years, and can find a recipe for anything I desire. It makes a wonderfully useful volume. I mean never to be without it.

Yours,

J. D. F.

Many letters in this strain could be published, showing the appreciation in which our magazine is held. Of course, it's a useful journal, else would we not have so large a circulation. The good people know a valuable thing when they see it.

---

CYNTHANA, KY., Dec. 1, 1879.

DEAR BISTOURY:

What has become of Fritz ? We have heard nothing from him in some time. We hope the BISTOURY has not dropped him for good. A sprightly boy as that must have done something worth recording by this time. Come, let us hear from him.

MRS. A. N.

Fritz nearly exhausted himself entertaining his friends in "BODINES." He is just now getting up steam for a new departure. There are external evidences that an eruption must soon transpire. We will probably keep our correspondent informed of what occurs.

---

WILLIAMSPORT, PA., Oct. 30, 1879.

DR. UP DE GRAFF :

I am almost afraid to ask you a question after your ill-natured reply to "Mamie" in the last BISTOURY. But I will just venture to ask you if you can give me a recipe for tan and freckles. Now, please don't give me a lecture on kitchen duties and all that, but give me a civil answer, now, please.

JENNIE.

To tell the truth, Jennie, you are not the first lady that has rebuked us for that ungracious reply, referred to. We are ashamed of it, and ask "Mamie's" pardon—there now. You will find the recipe inquired for in the department of Medical Items and News.

---

ROCHESTER, N. Y., Dec. 1, 1879.

DEAR BISTOURY:

You seem to know almost everything ; can you tell me how to make a hektograph ? They seem to be simple enough, yet are so very costly when purchased in the stores.

D.

Procure the following ingredients:

Pure glycerine, 20 oz.
Pure dextrine, 7 dr.
Sulphate baryta, 9 oz.
Gelatine, 13 dr.

All by weight, not liquid measure.

Place the baryta in a bright tin pan and stir in the glycerine, heating it over the stove. Cover the gelatine with *cold* water and heat it also (in another dish) until entirely melted. Dissolve the dextrine in hot water and stir in a pan, over the fire, until melted. Now pour all together in one of those long, square, tin pie pans. You now have a pad about 7x12 inches that will reproduce your letter up to forty or fifty copies better than any pad sold in the shops. Hektograph ink, to be found at the bookstores, is used. Wash your pad with luke-warm water and soft sponge as soon as the printing is over, else will the ink sink into the pad so deep as to prevent removal. Press a sheet of blotting paper over the pad before using it the first time.

If the mixture is strained through a cloth, it makes its surface more smooth when cool. Have your pan level.

